# Quantitative criteria characterizing the time change pattern of total lipid-peroxidation carbonyls

**DOI:** 10.1038/s41598-022-27066-1

**Published:** 2022-12-26

**Authors:** Reza Farhoosh

**Affiliations:** Department of Food Science and Technology, Ferdowsi University of Mashhad, Faculty of Agriculture, P.O. Box 91775-1163, Mashhad, Iran

**Keywords:** Natural hazards, Chemistry, Mathematics and computing

## Abstract

The present study introduced for the first time a sigmoidal function regarding the change in lipid-peroxidation carbonyls over time, providing a number of helpful kinetic parameters to more reliably evaluate the secondary oxidation of lipid systems. The sigmoidal function was of excellent goodness of fit (*R*^2^ > 0.99) in the non-inhibited and inhibited (in the presence of the antioxidant TBHQ) fatty acid compositions (sunflower and olive oils) at 80 and 100 °C. The calculated kinetic parameters were able to significantly differentiate among the oxidizing systems exposed to the experimented intrinsic/extrinsic factors. The new methodology developed in this study enabled us to more quantitatively indicate the resistance of lipid systems to the production of one of the most important destructive compounds from nutritional and sensory standpoints.

## Introduction

**Figure 1:**
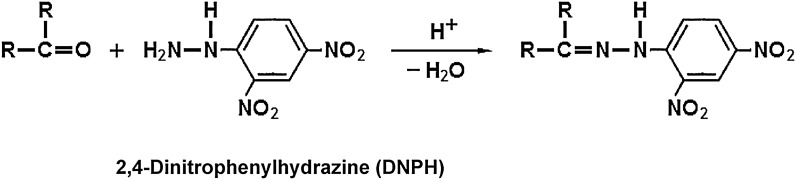
The reaction between carbonyls and 2,4-dinitrophenylhydrazin (DNPH) forming the corresponding 2,4-DNPhydrazones.

Lipid-peroxidation carbonyls (LCO), which is mainly of aldehydic or ketonic nature, are the most reactive secondary oxidation products arising from the decomposition of preformed lipid hydroperoxides (LOOH). The formation of LCO is of great concern for many lipid scientists, due to their destructive impact on the nutritional value and sensory properties of lipid systems. The most widely used method to determine total carbonyl compounds (known as carbonyl value, CV) in oxidized lipids, including saturated/unsaturated^1^ and volatile/non-volatile^2^ carbonyls, is the procedure reported by Henick et al.^3^, which is still used today but with some modifications^1,4,5^. The method involves the reaction of LCO with 2,4-dinitrophenylhydrazine (DNPH) to form colored 2,4-DNPhydrazones being spectrophotometrically measurable in an alkaline solution (Fig. 1).

During autoxidation, the total content of LCO increases slowly followed by a rapid rise corresponding to the decomposition of LOOH attained their maximum value^6^ into carbonyl compounds^7^. After reaching their maximum value, LCO show constant or reduced concentrations^2,8–11^. This might be due to the further degradations of carbonyls to some new products of lower molecular weight, which may escape from the oxidizing system, or of non-carbonyl character, which are not detectable by the CV assay^2,9^. Under the more advanced stages of lipid peroxidation, saturated aldehydes have been shown to undergo easy oxidation and dimerization/condensation reactions, providing a range of non-aldehydes. Also, unsaturated aldehydes are further oxidized into more volatile aldehydes/dialdehydes, short-chain hydrocarbons, and mono- and dibasic acids^12^.

From a mathematical point of view, the change in the LCO content exhibits a sigmoidal pattern. Such a pattern may provide some quantitative criteria for more reliable evaluations of the secondary oxidation of lipid systems. Sigmoidal equations essentially possess a turning point at which the rate of change in the y-coordinate reaches a maximum value. The equations also tend asymptotically to a finite value, where the rate of change approaches zero at infinity. The time required to reach the finite value may provide some valuable information on the oxidative stability of the corresponding lipid system^13^. In the same manner, the duration in which an oil has a CV ≤ 43.5 μmol g^−1^ (*t*_43.5_) has been known as the safety time range of nutritional and sensory interest^9^.

Literature shows no kinetic models describing quantitatively the change in the content of LCO over time. Hence, the present study aimed to mathematically formulate the time change pattern of LCO content during lipid peroxidation (see the section "Kinetic parameters derived from the LCO accumulation curves") and to provide some LCO-based quantitative criteria to more reliably evaluate the oxidative stability of lipid systems. To investigate how the sigmoidal equation works, LCO was monitored in the two purified edible oils (sunflower and olive) of quite different composition of fatty acids at 80 °C. Moreover, the time change pattern of LCO in the two oils was studied as affected by the antioxidant *tert*-butylhydroquinone (TBHQ) or a higher temperature at 100 °C.

## Materials and methods

### Materials

Commercially refined, bleached, and deodorized sunflower (SO) and olive (OO) oils were purchased from a local market. The oil samples were stored at − 18 °C until analysis. All chemicals and solvents used in this study were of analytical reagent grade and purchased from Merck (Darmstadt, Germany) and Sigma-Aldrich (St. Louis, MO).

### Purification of the oils

Chromatographic glass columns (40 × 3.5 cm I.D.) were packed by aluminium oxide 60 (active, neutral, 120 g) activated at 240 °C for 4 h right before use. The columns and collection vessels were wrapped in aluminium foil, and the oils were drawn through the column by suction without solvent. To ensure complete purification of the oils, the contents of phenolics (according to the Folin–Ciocalteau procedure described by Capannesi et al.^14^), tocopherols (according to the colorimetric method described by Wong et al.^15^), and hydroperoxides (according to the spectrophotometric method described by Shantha and Decker^16^) were measured in the purified oils.

### Fatty acid composition

Fatty acids were transesterified into methyl esters by vigorous shaking of a solution of oil in *n*-hexane (0.3 g in 7 mL) with 2 mL of 7 N methanolic potassium hydroxide at 50 °C for 10 min. The methyl esters were identified using an HP-5890 gas chromatograph (Hewlett-Packard, CA, USA) equipped with a CP-FIL 88 (Supel Co., Inc., Bellefonte, PA) capillary column of fused silica, 60 m in length × 0.22 mm I.D., 0.2 µm film thickness, and a flame ionization detector. Nitrogen was used as carrier gas with a flow rate of 0.75 mL min^−1^. The temperature of oven, injector and detector was maintained at 198, 250 and 250 °C, respectively. Standard fatty acid methyl esters from Merck (Darmstadt, Germany) were used for identification purposes^17^.

### Oxidation

The 1-mm layers of the oils (4 g) in Petri dishes of 9 cm in diameter were stored in a dry ventilated oven set at 80 and 100 °C. The oils containing 1.2 mM of TBHQ were prepared by adding aliquots of their solutions in acetone. The acetone was removed under a steam of nitrogen. The oil samples (0.04–1.0 g) were withdrawn at certain time intervals and subjected to the spectrophotometric determination of the amount (μmol g^−1^) of LCO (CV, see below).

### CV measurement

2-Propanol containing 0.05% (w/w) of sodium borohydride was refluxed for 1 h and then distilled to remove any trace of carbonyl compounds. 2,4-DNPH (50 mg) was dissolved in 100 mL of 2-propanol containing 3.5 mL of 37% HCl. Oil samples (0.04–1.0 g) were dissolved in 10 mL of 2-propanol containing triphenylphosphine (0.4 mg mL^−1^) to reduce hydroperoxide formation. 2,4-Decadienal in 2-propanol (50–500 μM) was used as the standard carbonyl. The standard/oil solutions (1 mL) were mixed with 1 mL of the DNPH solution in a 15-mL test tube. The stoppered test tubes were heated for 20 min at 40 °C. They were cooled in water, and 2% KOH solutions (8 mL) were added. The test tubes were centrifuged at 2000 × *g* for 5 min at room temperature. The absorbance of the upper layers were read at 420 nm against a blank containing all the reagents except that the standard carbonyl solution or the oil was replaced by the solvent alone^1,5^.

### Kinetic parameters derived from the LCO accumulation curves

**Figure 2:**
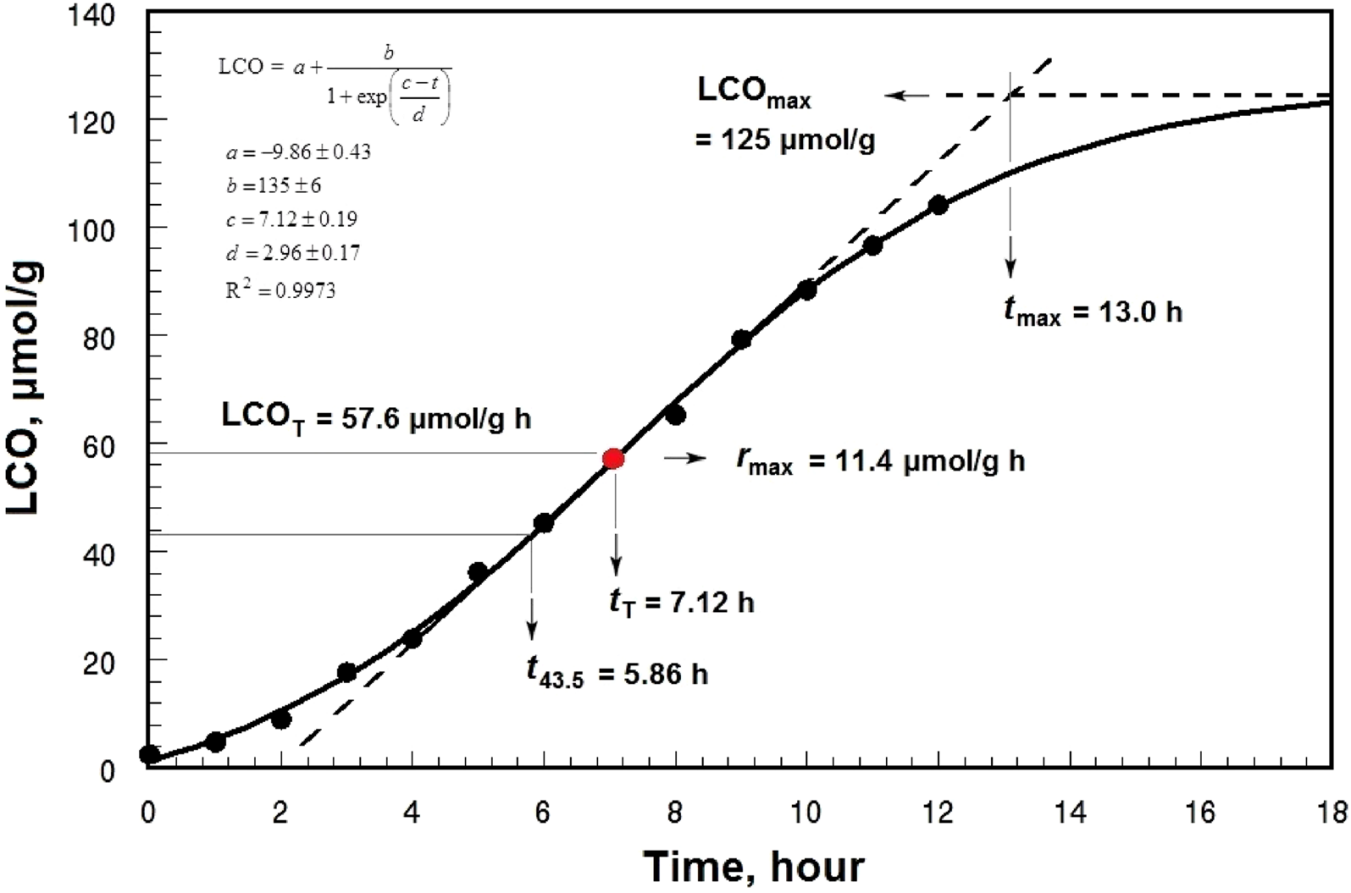
Kinetic curve of the accumulation of lipid-peroxidation carbonyls (LCO) during peroxidation of the sunflower oil at 80 °C, and the kinetic parameters from the sigmoidal equation fitted on the whole range of the data points. *t*_43.5_: the time required to reach a LCO content of 43.5 μmol g^−1^; LCO_T_ and *t*_T_: the coordinates of the turning point at which the rate of LCO accumulation reaches the maximum value *r*_max_; LCO_max_: the maximum concentration of LCO attained during the course of lipid peroxidation; *t*_max_: the time required to reach LCO_max_.

Kinetic curves of LCO accumulation were drawn by plotting the changes in CV versus time *t* (h) (Fig. 2). The sigmoidal Eq. (1) was fitted on the kinetic data points of LCO accumulation:

1 $${\text{[LCO] = }}a + \frac{b}{{1 + \exp \left( {\frac{c - t}{d}} \right)}}$$

where *a*, *b*, *c*, and *d* are the parameters of the sigmoidal equation. The equation asymptotically tends to the finite value LCO_max_, where the rate of LCO accumulation reaches zero at infinity:

2 $${\text{LCO}}_{{{\text{max}}}} {\text{ = lim}}_{t \to \infty } \left\{ {a + \frac{b}{{1 + \exp \left( {\frac{c - t}{d}} \right)}}} \right\} = a + b$$

The sigmoidal Eq. (1) exhibits a turning point at which the rate of LCO accumulation reaches the maximum value *r*_max_ (μmol g^−1^ h^−1^). The second derivative (Eq. 3) of Eq. (1) at *t* = 0 gives the coordinates (*t*_T_ and LCO_T_) of the turning point:

3 $$\frac{{d^{2} {\text{LCO}}}}{{dt^{2} }}{ = }\frac{{b\exp \left( {\frac{c + t}{d}} \right)\left\{ {\exp \left( \frac{c}{d} \right) - \exp \left( \frac{t}{d} \right)} \right\}}}{{d^{2} \left\{ {\exp \left( \frac{c}{d} \right) + \exp \left( \frac{t}{d} \right)} \right\}^{3} }}$$

4 $$t_{{\text{T}}} { = }c$$

5 $${\text{LCO}}_{{\text{T}}} { = }a + \frac{b}{2}$$

Substituting *t* in the first derivative (Eq. 6) of Eq. (1) by *t*_T_ from Eq. (4) provides the value of *r*_max_:

6 $$\frac{{d{\text{LCO}}}}{dt}{ = }\frac{{b\exp \left( {\frac{c - t}{d}} \right)}}{{d\left\{ {1 + \exp \left( {\frac{c - t}{d}} \right)} \right\}^{2} }}$$

7 $$r_{\max } = \left( {\frac{{d{\text{LCO}}}}{dt}} \right)_{{{\text{max}}}} { = }\frac{b}{{{4}d}}$$

The ratio between *r*_max_ and LCO_max_ can be considered as the normalized form (*r*_n_, h^−1^) of the maximum rate of LCO accumulation:

8 $$r_{{\text{n}}} { = }\frac{{r_{{{\text{max}}}} }}{{{\text{LCO}}_{{{\text{max}}}} }} = \frac{b}{{4d\left( {a + b} \right)}}$$

The linear Eq. (9), resulting from the slope (Eq. 7) and coordinates of the turning point (Eqs. 4 & 5), at LCO = LCO_max_ gives the duration (*t*_max_, h) at which carbonyls approach their maximum value, LCO_max_.

9 $${\text{LCO}} = r_{{{\text{max}}}} \left( {t - t_{{\text{T}}} } \right) + {\text{LCO}}_{{\text{T}}}$$

10 $$t_{\max } = \frac{{{\text{LCO}}_{{{\text{max}}}} - {\text{LCO}}_{{\text{T}}} + \left( {r_{{{\text{max}}}} . \, t_{{\text{T}}} } \right)}}{{r_{{{\text{max}}}} }} = c + 2d$$

The value of *t*_43.5_ (h) can be calculated from Eq. (1) at LCO = 43.5 as follows:

11 $$t_{43.5} = c - d\ln \left( {\frac{a + b - 43.5}{{43.5 - a}}} \right)$$

### Statistical analysis

All determinations were carried out in triplicate and data were subjected to analysis of variance (ANOVA). ANOVA and regression analyses were performed according to the MStatC and SlideWrite software version 7.0. Significant differences between means were determined by Duncan’s multiple range tests. P values less than 0.05 were considered statistically significant.

## Results and discussion

### Chemical composition of the oil samples

The purified oils contained no detectable LOOH, tocopherols, and phenolic compounds, indicating suitable performance of the purification process. The fatty acid composition of the oils was in agreement with those usually reported for SO and OO ^18^ (Table 1). OO was of significantly higher amount of saturated fatty acids (SFA, mainly palmitic acid, C16:0) than SO. OO was constituted of almost threefold amount of monounsaturated fatty acids (MUFA, mainly oleic acid, C18:1) compared to SO. However, a sixfold amount of polyunsaturated fatty acids (PUFA, mainly linoleic acid, 18:2) was found in SO. Considering the relative rate of oxidation for C18:3, C18:2, C18:1, and C18:0 as 2500:1200:100:1

**Table 1:** Fatty acid composition (%w/w) of the vegetable oils studied.

Fatty acid	Oil sample	
	Sunflower	Olive
C14:0	0.05 ± 0.00	–
C16:0	6.73 ± 0.03^b^	12.7 ± 0.7^a^
C16:1	0.08 ± 0.00^b^	0.91 ± 0.01^a^
C18:0	4.28 ± 0.08^a^	3.23 ± 0.01^b^
C18:1n-9	24.3 ± 0.4^b^	71.2 ± 0.9^a^
C18:2n-6	62.4 ± 0.3^a^	9.6 ± 0.2^b^
C18:3n-3	0.24 ± 0.00^b^	0.64 ± 0.00^a^
C20:0	0.25 ± 0.00^b^	0.38 ± 0.00^a^
C20:1	0.13 ± 0.00^b^	0.35 ± 0.00^a^
C20:2	0.06 ± 0.00	–
C22:0	0.81 ± 0.05^a^	0.14 ± 0.00^b^
C22:1	0.04 ± 0.00^b^	0.55 ± 0.00^a^
C24:0	0.28 ± 0.00^a^	0.16 ± 0.00^b^
SFA	12.4 ± 0.4^b^	16.6 ± 0.4^a^
MUFA	24.6 ± 0.3^b^	73.0 ± 0.6^a^
PUFA	62.6 ± 0.2^a^	10.2 ± 0.1^b^

Means ± SD (standard deviation) within a row with the same lowercase letters are not significantly different at *p* < 0.05. SFA: Saturated fatty acids; MUFA: Monounsaturated fatty acids; PUFA: Polyunsaturated fatty acids.

^19^, it was expected that SO to be of markedly lower oxidative stability than OO.

### Kinetic data analysis

Figure 2 shows the kinetic curve of LCO accumulation during peroxidation of SO at 80 °C. As can be seen, the sigmoidal Eq. (1) could appropriately fit the change in the LCO content over time. Such a pattern with coefficients of determination (R^2^) higher than 0.99 was also obtained for the other oxidation treatments. Table 2 presents the quantitative criteria characterizing the time change pattern of LCO during peroxidation of SO and OO as affected by temperature and the antioxidant TBHQ.

**Table 2:** The parameters resulting from the sigmoidal equation fitted on the kinetic curves of the accumulation of lipid-peroxidation carbonyls (LCO) during oxidation of the sunflower (SO) and olive (OO) oils in the presence and absence of *tert*-butylhydroquinone (TBHQ) at 80 and 100 °C.

Parameter	80 °C				100 °C	
	SO	OO	SO + TBHQ	OO + TBHQ	SO	OO
LCO_T_ (μmol g^−1^)	57.6 ± 1.8^c^	50.0 ± 0.0^d^	233 ± 11^a^	67.7 ± 1.0^b^	51.5 ± 1.5^d^	57.3 ± 0.4^c^
LCO_max_ (μmol g^−1^)	125 ± 5^b^	94.9 ± 1.7^e^	464 ± 21^a^	130 ± 1^b^	106 ± 2^d^	111 ± 1^c^
*t*_T_ (h)	7.12 ± 0.19^e^	21.3 ± 0.1^c^	179 ± 2^b^	252 ± 1^a^	2.38 ± 0.03^f^	8.46 ± 0.05^d^
*t*_max_ (h)	13.0 ± 0.5^d^	27.9 ± 0.0^c^	239 ± 3^b^	335 ± 1^a^	3.93 ± 0.11^f^	12.0 ± 0.0^e^
*t*_43.5_ (h)	5.86 ± 0.02^e^	20.4 ± 0.2^c^	111 ± 1^b^	218 ± 2^a^	2.15 ± 0.02^f^	7.52 ± 0.10^d^
*r*_max_ (μmol g^−1^ h^−1^)	11.4 ± 0.1^c^	6.79 ± 0.05^d^	3.90 ± 0.13^e^	0.75 ± 0.01^f^	35.0 ± 1.6^a^	15.1 ± 0.2^b^
*r*_n_ (h^−1^)	0.0913 ± 0.0045^c^	0.0716 ± 0.0007^d^	0.0084 ± 0.0001^e^	0.0058 ± 0.0001^f^	0.3309 ± 0.0143^a^	0.1360 ± 0.0030^b^

Means ± SD (standard deviation) within a row with the same lowercase letters are not significantly different at *P* < 0.05. *LCO*_*T*_ LCO concentration at the turning point of the sigmoidal equation; *LCO*_max_ The maximum concentration of LCO attained during the course of lipid peroxidation; *t*_T_ The time required to reach LCO_T_; *t*_max_ The time required to reach LCO_max_; *t*_*43.5*_ The time required to reach a LCO content of 43.5 μmol g^−1^; *r*_max_ Maximum rate of LCO accumulation; *r*_n_ Normalized *r*_max_.

At 80 °C, SO appeared a LCO_max_ value of significantly higher than that of OO (Table 2). This can be attributed to the higher rate of LCO accumulation, which is represented by the value of *r*_max_, in SO with the fatty acid composition (Table 1) of lower resistance to lipid peroxidation. This was also consistent with the significantly lower value of *r*_n_, which unifies the two parameters, for OO than for SO. The time parameters, especially the safety time range *t*_43.5_, demonstrated the much longer time of application for OO by a factor of 3.5.

Peroxidation at the higher temperature provided some other interesting results. As shown in Table 2, the value of LCO_max_ for SO significantly decreased from 125 μmol g^−1^ at 80 °C to 106 μmol g^−1^ at 100 °C. This was while the value of *r*_max_ at 80 °C (11.4 μmol g^−1^ h^−1^) underwent an increase of about threefold to 35.0 μmol g^−1^ h^−1^ at 100 °C. Such an occurrence could be explained by the higher degradation rate of carbonyls to the fragments of lower molecular weight, escaping more easily from the system, or to non-carbonyls undetectable by the CV assay^2,9^. By contrast with SO, the rise of temperature from 80 to 100 °C caused a significant increase in the value of LCO_max_ in OO from 94.9 to 111 μmol g^−1^, although the value of *r*_max_ with an increase of about 2.2-fold changed from 6.79 to 15.1 μmol g^−1^ h^−1^. This can be attributed to the lower tendency of OO’s carbonyls to form degraded products, which is also considered in the values of *r*_n_ (0.3309 vs. 0.1360 h^−1^). Besides, the other quantitative criterion *t*_43.5_ demonstrated as well the higher oxidative stability of OO (7.52 h) than SO (2.15 h) at 100 °C.

The powerful synthetic antioxidant TBHQ caused LCO_max_ values at 80 °C to increase dramatically in SO by a factor of 3.7 and significantly in OO by a factor of 1.4 (Table 2). At the same time, the value of *r*_max_ in SO and OO considerably decreased by factors of 2.9 and 9.1, respectively. This clearly shows the relatively greater capability of TBHQ in preventing the LCO degradation in SO but its fairly better potency in inhibiting the formation of LCO in OO. On the whole, the antioxidant was able to lower the values of *r*_n_ from 0.0913 to 0.0084 h^−1^ in SO and from 0.0716 to 0.0058 h^−1^ in OO. In a practical term, the safety time range *t*_43.5_ in SO and OO increased by factors of about 19 and 11, respectively, implying higher antioxidant efficiency of TBHQ in the lipid system of lower resistance to the formation of carbonyl compounds.

## Conclusions

This study could mathematically formulate the time change pattern of lipid-peroxidation carbonyls (LCO) as the most reactive secondary oxidation products of intensively destructive impact on the nutritional value and sensory properties of lipid systems. The time change pattern of LCO as affected by some of the most important variables influencing oxidative stability, including fatty acid composition, temperature, and inhibitor, which could naturally encompass wider ranges than those examined in the present study, followed a sigmoidal function with excellent coefficients of determination (R^2^ > 0.99). The sigmoidal function gave us many valuable kinetic parameters, especially LCO_max_, *r*_max_, *r*_n_, and *t*_43.5_, to reliably quantitate the resistance of the oxidizing media to the production of carbonyl compounds. On this basis, researchers of the field are provided with a new insight in their future studies into the quantitative investigation on the secondary oxidation of lipid systems as a function of any intrinsic and/or extrinsic factor.

## Data Availability

All the necessary data generated and/or analysed during the current study are included in this published article and its additional information, if needed, are available from the corresponding author on reasonable request.
